# Comparison of functional residual capacity and static compliance of the respiratory system during a positive end-expiratory pressure (PEEP) ramp procedure in an experimental model of acute respiratory distress syndrome

**DOI:** 10.1186/cc6961

**Published:** 2008-07-16

**Authors:** Bernard Lambermont, Alexandre Ghuysen, Nathalie Janssen, Philippe Morimont, Gary Hartstein, Paul Gerard, Vincent D'Orio

**Affiliations:** 1 Hemodynamic Research Center, HemoLiege, University of Liege, Belgium; 2 Medical Intensive Care Unit, Department of Medicine, University Hospital of Liege, Belgium; 3 Emergency Care Department, University Hospital of Liege, Belgium; 4 Department of Statistics, University of Liege, Belgium

## Abstract

**Introduction:**

Functional residual capacity (FRC) measurement is now available on new ventilators as an automated procedure. We compared FRC, static thoracopulmonary compliance (Crs) and PaO_2 _evolution in an experimental model of acute respiratory distress syndrome (ARDS) during a reversed, sequential ramp procedure of positive end-expiratory pressure (PEEP) changes to investigate the potential interest of combined FRC and Crs measurement in such a pathologic state.

**Methods:**

ARDS was induced by oleic acid injection in six anesthetised pigs. FRC and Crs were measured, and arterial blood samples were taken after induction of ARDS during a sequential ramp change of PEEP from 20 cm H_2_O to 0 cm H_2_O by steps of 5 cm H_2_O.

**Results:**

ARDS was responsible for significant decreases in FRC, Crs and PaO_2 _values. During ARDS, 20 cm H_2_O of PEEP was associated with FRC values that increased from 6.2 ± 1.3 to 19.7 ± 2.9 ml/kg and a significant improvement in PaO_2_. The maximal value of Crs was reached at a PEEP of 15 cm H_2_O, and the maximal value of FRC at a PEEP of 20 cm H_2_O. From a PEEP value of 15 to 0 cm H_2_O, FRC and Crs decreased progressively.

**Conclusion:**

Our results indicate that combined FRC and Crs measurements may help to identify the optimal level of PEEP. Indeed, by taking into account the value of both parameters during a sequential ramp change of PEEP from 20 cm H_2_O to 0 cm H_2_O by steps of 5 cm H_2_O, the end of overdistension may be identified by an increase in Crs and the start of derecruitment by an abrupt decrease in FRC.

## Introduction

In acute respiratory distress syndrome (ARDS) the setting of positive end-expiratory pressure (PEEP) is determined using several methods, including FiO_2 _requirement, measurement of either static (Crs) or dynamic thoracopulmonary compliance [1-4], generation of pressure-volume curves [2,5,6] and, rarely, using computed tomography (CT) scan analysis [5,7-10]. During a decremental PEEP manoeuvre, the point of maximal Crs has been shown to correspond to the minimum open lung positive end-expiratory pressure preventing end-expiratory collapse of those alveoli which are inflated at end inspiration [1]. The volume recruited by PEEP is usually assessed by a method based on the static pressure-volume curve of the respiratory system. Alveolar recruitment leads to an upward shift along the volume axis of the pressure-volume curve with PEEP, compared to the curve with zero end-expiratory pressure, and is quantified as the volume increase with PEEP at the same elastic pressure [11]. Functional residual capacity (FRC), which reflects the amount of gas present in the lungs, has been suggested to be a better indicator than Crs to assess the state of recruitment and derecruitment caused by PEEP manipulations, because it directly measures the lung volume increase induced by PEEP, mainly due to the recruitment of collapsed alveoli [12]. However, FRC measurement is not usually performed at the bedside because of technical limitations. More recently, an automated procedure for FRC measurement has become available and is incorporated into the software of specific intensive care ventilators [13]. Therefore, we compared FRC, Crs and PaO_2 _evolution in an experimental model of ARDS during a reversed sequential ramp procedure of PEEP changes to investigate the potential interest of combining FRC and Crs measurements in such a pathologic state.

## Materials and methods

All experimental procedures and protocols used in this investigation were reviewed and approved by the Ethics Committee of the Medical Faculty of the University of Liege. The investigation conforms with guidelines on laboratory animals published by the US National Institutes of Health.

Six pigs weighing 26 ± 2 kg were premedicated with tiletamin/zolazepam 5 mg/kg and subsequently anaesthetised by a continuous infusion of sufentanil 0.5 μg/kg/h, pentobarbital 5 mg/kg/h and cisatracurium 2 mg/kg/h. They were ventilated through a tracheotomy in volume control mode at a fraction of inspired oxygen (FiO_2_) of 0.5 with a tidal volume of 10 ml/kg, an inhalation/exhalation (I:E) ratio of 1:2, a rate of 20 breath/min, and 5 cm H_2_O PEEP (Engström CareStation, Datex, General Electric, Finland).

Systemic arterial pressure was measured by a catheter (Sentron pressure-measuring catheter, Cordis, Miami, FL, USA) introduced in the abdominal aorta through the right femoral artery. Heart rate was obtained from one derivation continuous electrocardiogram monitoring.

FRC was calculated using an automated procedure available on the ventilator based on the nitrogen washout method with a FiO_2 _step change of 0.1, as previously described by Olegard *et al*. [13]. Using sidestream gas analysing technology, calculation of FRC values was obtained by applying the following equations.

The fractions of inspired and end-tidal nitrogen were calculated from:

F_I_N_2 _= 1 - F_I_O_2_

ETN_2 _= 1 - ETO_2 _- ETCO_2_

Expired and inspired alveolar tidal volumes were calculated using energy expenditure measurements for VO_2 _and VCO_2 _where VO_2 _= (VCO_2_/RQ):

$${\text{V}}_{\text{t}}^{\text{alv}(\text{E})}=\frac{{\text{VCO}}_{\text{2}}}{{\text{ETCO}}_{\text{2}}\text{.RR}}$$

$${\text{V}}_{\text{t}}^{\text{alv}(\text{I})}={\text{V}}_{\text{t}}^{\text{alv}(\text{E})}+\frac{{\text{VO}}_{\text{2}}\;-{\text{VCO}}_{\text{2}}}{\text{RR}}$$

Nitrogen volumes associated with expiration and inspiration for a single breath were:

$${\text{V}}_{\text{E}}^{{\text{N}}_{2}}={\text{ETN}}_{2}.{\text{V}}_{\text{t}}^{\text{alv}(\text{E})}$$

$${\text{V}}_{\text{I}}^{{\text{N}}_{2}}={\text{F}}_{\text{I}}{\text{N}}_{2}.{\text{V}}_{\text{t}}^{\text{alv}(\text{I})}$$

The changes during one breath equalled:

$$\Delta {\text{V}}^{{\text{N}}_{2}}={\text{V}}_{\text{E}}^{{\text{N}}_{2}}-{\text{V}}_{\text{I}}^{{\text{N}}_{2}}$$

Before making the step change in F_I_O_2_, a baseline condition was determined. This involved the determination of VO_2_, VCO_2 _and ETN_2_^baseline^. VO_2 _anv VCO_2 _were assumed to be constant throughout the measurement. After a step response the FRC was calculated as:

$$\text{FRC}=\frac{\Delta {\text{V}}^{\text{N}2}}{\Delta {\text{ETN}}_{2}}$$

Where the ETN_2 _was the last recorded value after the step change:

$$\text{FRC}=\frac{\sum_{\text{breaths}}\Delta {\text{V}}^{{\text{N}}_{2}}}{{\text{ETN}}_{2}^{\text{baseline}}-{\text{ETN}}_{2}}$$

Airway pressure values were measured by the ventilator at the level of the Y piece just before the tracheostomy tube. Crs was measured by holding a 10 s inspiratory pause to obtain the value of the inspiratory plateau airway pressure (Pins) and a 10 s expiratory pause to obtain the end-expiratory airway pressure (Pexp). The value of Crs was obtained by dividing tidal volume (Vt) by the difference between inspiratory plateau airway pressure and end-expiratory airway pressure:

Crs = Vt/(Pins - Pexp)

## Materials and methods

After a 30-min period of stabilisation, measurements were obtained at a PEEP of 5 cm H_2_O (basal). Then, ARDS was induced by administration of 0.12 ml/kg of oleic acid over 30 min.

At 120 min after the beginning of oleic acid injection, a set of parameters was obtained at a PEEP level of 5 cm H_2_O (ARDS). Subsequently, PEEP was increased to 20 cm H_2_O and then reduced by steps of 5 cm H_2_O to 0 cm H_2_O (ARDS20, ARDS15, ARDS10, ARDS5, ARDS0). Each PEEP level was maintained for 15 min before a set of measurements to allow for haemodynamic and respiratory stabilisation.

Arterial blood samples were taken during the basal condition (basal), 120 min after oleic acid injection, and at each PEEP level during ARDS.

Animals received neither vasoactive nor inotrope drugs during the procedure.

### Statistics

Data are presented as mean ± standard error of the mean. Statistical comparison of data over time was conducted by a two-way analysis of variance (ANOVA) for repeated measurements, followed by Scheffe's multiple comparisons test if the analysis of variance resulted in p value < 0.05 (Statistica, Statsoft Inc., Tulsa, OK, USA).

Correlations between FRC, static compliance, and PaO_2 _were evaluated by a Pearson's linear correlation test (Statistica). Difference between correlations was evaluated by an equality of dependent correlations test [14]. A p value < 0.05 was considered statistically significant.

## Results

ARDS was responsible for significant decreases in both FRC and Crs, from 16 ± 2 to 6.2 ± 1.3 ml/kg and from 28 ± 2 to 17 ± 1 ml/cm H_2_O, respectively. Values of PaO_2 _changed from 201 ± 7 to 52 ± 5 mmHg 120 min after oleic acid administration.

**Figure 1:**
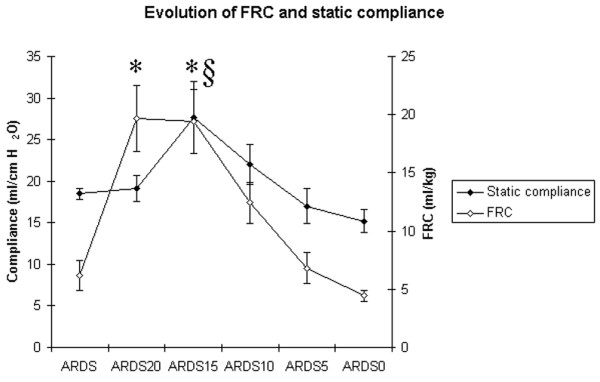
Time course of functional residual capacity (FRC) and static compliance of the respiratory system (Crs) during a decremental positive end-expiratory pressure (PEEP) trial after acute respiratory distress syndrome (ARDS) induction by oleic acid injection. Measurements were obtained 120 min after the oleic acid injection (ARDS) at a PEEP of 5 cm H_2_O and during a decremental PEEP trial from 20 to 0 cm H_2_O by steps of 5 cm H_2_O (ARDS20, ARDS15, ARDS10, ARDS5, ARDS0). Each PEEP level was maintained for 15 min before a set of measurements to allow for haemodynamic and respiratory stabilisation. § p < 0.05 vs ARDS (Crs); * p < 0.05 vs ARDS (FRC).

During ARDS, 20 cm H_2_O of PEEP was associated with FRC values that increased from 6.2 ± 1.3 to 19.7 ± 2.9 ml/kg. This change was associated with a significant improvement in PaO_2_, which reached 172 ± 15 mmHg. The point of maximum Crs during ARDS was reached at a PEEP of 15 cm H_2_O. From a PEEP value of 15 to 0 cm H_2_O, FRC and Crs decreased progressively (Figure 1).

The time course of haemodynamic data, arterial blood gases, and inspiratory plateau airway pressure during ARDS is presented in Table 1. Inspiratory plateau pressure increased significantly (p < 0.05) from 14 ± 0.8 (basal) to 20 ± 0.5 cm H_2_O (ARDS) after oleic acid injection. During ARDS, inspiratory plateau airway pressure was significantly increased at a PEEP of 20 and 15 cm H_2_O (p < 0.05). Maximal oxygenation was obtained at a PEEP of 20 and 15 cm H_2_O. PaCO_2 _was 42 ± 2 mmHg during basal condition and 54 ± 5 mmHg after oleic acid injection. During ARDS, PaCO_2 _increased significantly from 54 ± 5 mmHg (ARDS) to 62 ± 3 at a PEEP of 0 cm H_2_O (p < 0.05).

**Table 1:** Time course of haemodynamic parameters, inspiratory plateau airway pressure, arterial blood pH, PaO_2 _and PaCO_2 _after oleic acid injection and during a decremental PEEP trial from 20 to 0 cm H_2_O.

	**HR (beats/min)**	**Mean AP (mmHg)**	**pH**	**PaO_2 _(mmHg)**	**PaCO_2 _(mmHg)**	**Inspiratory plateau pressure (cm H_2_O)**
ARDS	129 ± 3	75 ± 16	7.32 ± 0.06	52 ± 5	54 ± 5	20 ± 1
ARDS20	132 ± 4	66 ± 13	7.33 ± 0.04	172 ± 15*	54 ± 3	36 ± 3*
ARDS15	134 ± 4	73 ± 13	7.38 ± 0.02	175 ± 17*	54 ± 2	26 ± 1*
ARDS10	145 ± 3*	75 ± 9	7.28 ± 0.05	89 ± 19*	56 ± 5	23 ± 2
ARDS5	165 ± 10*	118 ± 9*	7.33 ± 0.02	52 ± 4*	59 ± 2	22 ± 2
ARDS0	153 ± 7*	101 ± 5*	7.3 ± 0.03	52 ± 4*	62 ± 3*	21 ± 2

Data are presented as mean ± standard error of the mean. Measurements were obtained 120 min after oleic acid injection at a PEEP of 5 cm H_2_O (ARDS) and during a decremential PEEP trial (ARDS20, ARDS15, ARDS10, ARDS5, ARDS0). Each PEEP level was maintained for 15 min before each measurement to allow for haemodynamic and respiratory stabilisation. ARDS, acute respiratory distress syndrome; HR, heart rate; mean AP, mean systemic arterial pressure; PEEP, positive end-expiratory pressure. * p < 0.05 versus ARDS

**Figure 2:**
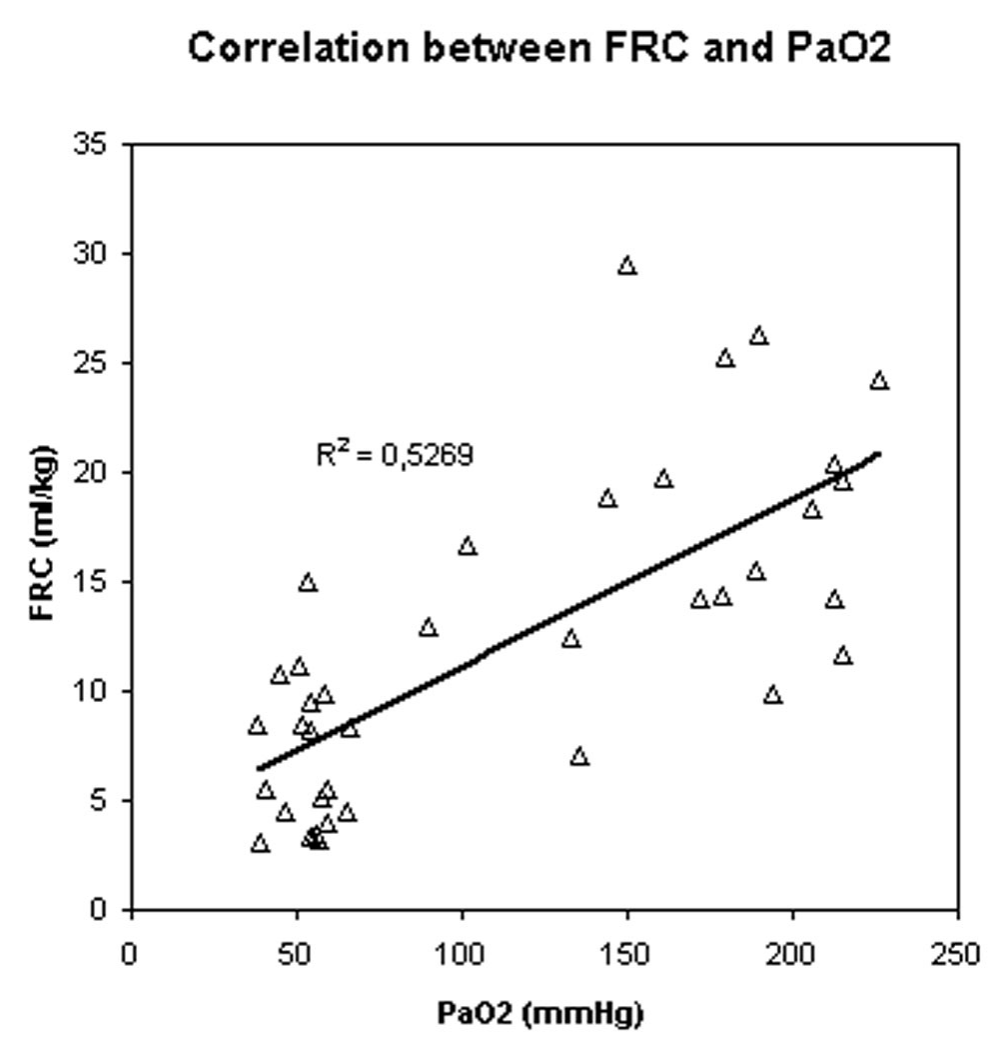
Correlation between PaO_2 _and functional residual capacity (FRC).

**Figure 3:**
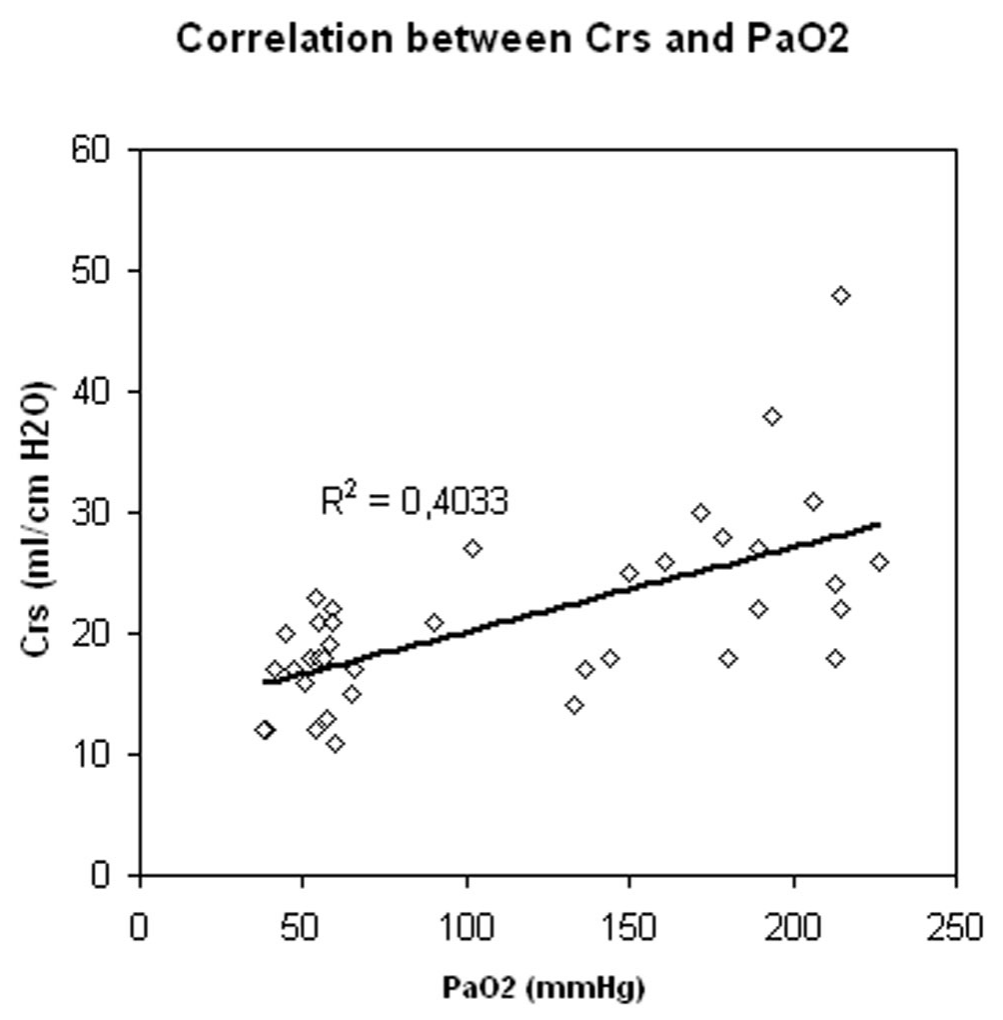
Correlation between PaO_2 _and static compliance of the respiratory system (Crs).

**Figure 4:**
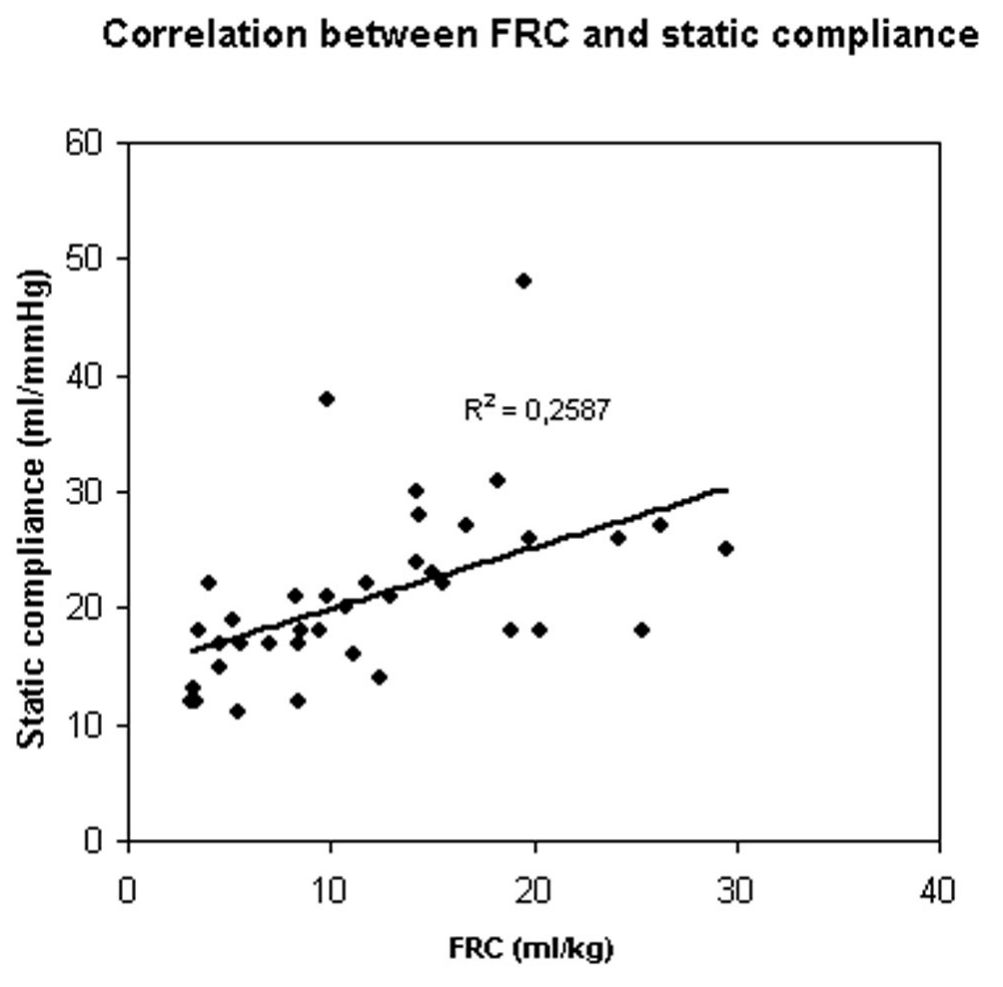
Correlation between functional residual capacity (FRC) and static compliance of the respiratory system (Crs).

Correlation between PaO_2 _and FRC (Figure 2), and between PaO_2 _and Crs (Figure 3) were significant (p < 0.05) but weak (r^2 ^= 0.53 and 0.4, respectively); the difference between the two correlations was not significant (p = 0.41). Correlation between FRC and Crs was also significant but weak (p < 0.05, r^2 ^= 0.26) (Figure 4).

## Discussion

In this experimental model of ARDS, FRC and Crs values obtained during mechanical ventilation were correlated to the changes in PaO_2 _obtained during a sequential ramp change of PEEP from 20 cm H_2_O to 0 cm H_2_O by steps of 5 cm H_2_O. The maximal value of Crs was reached immediately before FRC began to decrease.

Our results are in accordance with those of Suarez-Sipman *et al*., who showed that maximal dynamic compliance of the respiratory system immediately preceded the beginning of alveolar collapse after lung recruitment as shown by computed tomography (CT) scan studies [4]. Previous studies have already demonstrated a strong and inverse correlation between arterial oxygenation and the amount of collapsed lung mass in multislice CT scans [15]. Rylander *et al*. suggested that FRC was a more sensitive indicator of PEEP-induced aeration and recruitment of lung tissue than Crs [12]. However, an increase in FRC may be due to alveolar recruitment, but may also be secondary to alveolar overdistension. To distinguish between these possibilities, use of thoracopulmonary compliance has been suggested. Indeed, a parallel increase in FRC and thoracopulmonary compliance suggests alveolar recruitment while a decrease in thoracopulmonary compliance together with increasing FRC would tend to indicate alveolar overdistension [16]. Our results strengthen this suggestion: the fact that FRC did not decrease, while Crs increased, when PEEP decreased from 20 and 15 cm H_2_O suggests that alveolar overdistension was present at a PEEP of 20 cm H_2_O.

In this study, the optimal level of PaO_2 _was obtained at a PEEP of 20 and 15 cm H_2_O. Because one of the goals of PEEP is to reach an arterial saturation greater than 90% at the lowest possible FiO_2_, the monitoring of arterial oxygenation could be considered as the unique gold standard for optimising PEEP. However, because alveolar recruitment and lung overinflation can be simultaneously observed in different parts of the lung, changes in PaO_2 _cannot be considered sensitive enough to detect the risk of ventilator induced lung injury [10]. Increasing the level of PEEP can be more harmful than beneficial since it will serve also to increase inflation of lung regions that are already open, increasing the stress and strain on these regions [7]. Two studies showed a significant positive correlation between PEEP-related recruitment and arterial oxygenation [8,17]. The correlations found in these two studies are unfortunately relatively weak, suggesting that arterial oxygenation cannot be used reliably to predict the amount of recruitment induced by a given level of PEEP [9]. Our results confirm these data, since correlations between either FRC or Crs and PaO_2 _are also significant and weak without a difference between the two correlations.

As suggested by our results, such an assessment is a valuable tool to help to identify the optimal level of PEEP. It can also be used for trend analysis, as a decrease in FRC can be the first sign of derecruitment and may help the clinician to understand the pathophysiological mechanism worsening blood oxygenation. Finally, this parameter might provide practical help in therapeutic decision making [18].

To our knowledge, this study is the first to measure FRC by using the automated procedure available on the Engstrom Care Station ventilator in a porcine model of ARDS. This method has been validated by Olegard *et al*. [13]. They have shown that FRC measurement with high precision can be obtained using a N_2 _multiple breath washout technique based on standard gas monitoring equipment and an FiO_2 _step change as little as 0.1. As the calculation of FRC is based on the values of VCO_2_, end-tidal O_2 _and end-tidal CO_2_, all these values need to be valid to result in acceptable results. The conditions that may cause invalid data include: rapid and/or irregular respiratory rates, large variations in tidal volumes, high fevers, agitation, neurological conditions that alter respiration. Constant breathing patterns are required to achieve valid VCO_2 _measurements; this was the case in the experimental conditions of the present study, but may not be warranted in the clinical setting.

It could be argued that the effects of PEEP changes observed during the ramp procedure were due to a lack of stability of this ARDS model. However, The porcine oleic acid model of ARDS used in this study has been extensively studied and used to represent the early, exudative phase of ARDS. We allowed a 120-min period of stabilisation after oleic acid injection before initiating the PEEP ramp procedure in order to provide a stable condition. This is more time than is actually necessary since stable conditions can be generally reached in this model after 30 to 60 min, according to several previous studies [12,19,20].

No specific evaluation of the FRC technique was performed in this study. FRC measurements were performed during a decremental PEEP trial without specific evaluation of the technique, which was out of the scope of the study. However, it would be interesting to determine, in a complementary study, how reproducible the measurement is, and how this method of determining FRC compares to other techniques for absolute lung volume measurements as well as with techniques that measures lung volume changes. Finally, since the findings of this study were obtained using volume control mode ventilation with a tidal volume of 10 ml/kg, the efficacy of this method remains to be demonstrated in other ventilatory modes (such as pressure control) and also with the lower tidal volumes usually used during ARDS.

## Conclusion

Our results indicate that a combination of FRC and Crs measurements obtained in this porcine model of ARDS may help to identify the optimal level of PEEP. Indeed, by taking into account the value of both parameters, during a sequential ramp change of PEEP from 20 cm H_2_O to 0 cm H_2_O by steps of 5 cm H_2_O, the end of overdistension may be identified by an increase in Crs, and the start of derecruitment by an abrupt decrease in FRC. Using this approach to find the best value of PEEP should allow for the tidal excursion to be positioned between derecruitment and overdistension on the pressure-volume curve.

## Key messages

● Functional residual capacity measurement is now available on new ventilators as an automated procedure.

● Combined measurement of thoracopulmonary static compliance and functional residual capacity may help to identify the optimal level of PEEP in ARDS.

## Abbreviations

ARDS = acute respiratory distress syndrome; Crs = static thoracopulmonary compliance of the respiratory system; FRC = functional residual capacity, PEEP = positive end-expiratory pressure; Pexp = expiratory plateau airway pressure; Pins = inspiratory plateau airway pressure; Vt = tidal volume.

## Competing interests

The authors declare that they have no competing interests.

## Authors' contributions

BL, AG, NJ, PM participated in the design of the study, and collected the data during the experiments. BL and PG analysed the data and performed the statistical analysis. VD participated in the design and the coordination of the study and helped to draft the manuscript. BL, AG, NJ, PM, GH, PG, VD have been involved in drafting the manuscript or revising it critically and have given final approval of the version to be published. All authors read and approved the final manuscript.
